# Investigation of the Pozzolanic Activity Improvement of Yellow Phosphorus Slag with Thermal Activation

**DOI:** 10.3390/ma16176047

**Published:** 2023-09-03

**Authors:** Xinyue Liu, Xiaoming Liu, Zengqi Zhang, Chao Wei, Qingsen Zeng, Yantian Li, Shanliang Ma

**Affiliations:** 1 State Key Laboratory of Advanced Metallurgy, University of Science and Technology Beijing, Beijing 100083, China; b20200134@xs.ustb.edu.cn; 2 School of Metallurgical and Ecological Engineering, University of Science and Technology Beijing, Beijing 100083, China; weichao0810@126.com (C.W.); g20208335@xs.ustb.edu.cn (Q.Z.); liyantian2@126.com (Y.L.); g20208295@xs.ustb.edu.cn (S.M.)

**Keywords:** yellow phosphorus slag, thermal activation, pozzolanic activity, compressive strength, alkali dissolution, polymerization degree

## Abstract

Yellow phosphorus slag (YPS) is a byproduct from the production of yellow phosphorus. It has potential pozzolanic activity and can be used as a supplementary cementitious material. However, the early strength of cement mortar decreases significantly with increasing YPS dosage, which restricts the utilization of YPS in cement and concrete. This study aimed to increase the pozzolanic activity of YPS ash by thermal activation. The strength method, alkali dissolution method and polymerization degree method were used to evaluate the effect of thermal activation at different temperatures on the pozzolanic activity of YPS ash. The results showed that YPS ash calcined at 800 °C helps to enhance the early strength because the fluorine in cuspidine (Ca_4_Si_2_O_7_F_2_) is insoluble, reducing the retarding effect on the mortar. The higher late strength of YPS ash calcined at 100 °C was due to the low polymerization degree of [SiO_4_]. The pozzolanic activity of YPS ash is positively correlated with the dissolution concentration of (Si + Al) and the compressive strength and negatively associated with the polymerization degree. This paper shows a possibility for the large-scale utilization of YPS.

## 1. Introduction

Yellow phosphorus slag (YPS) is a byproduct from the production of yellow phosphorus using phosphorus ore by the electric furnace method [1,2]. Phosphate ore reacts with coke to form phosphorus, and its impurities react with silica to obtain YPS [3]. Each ton of yellow phosphorus will produce approximately 8–12 tons of YPS [4]. China, as the largest yellow phosphorus producer, produces approximately 8 million tons of YPS annually, but the comprehensive utilization rate of YPS is lower than 50% [4]. Most YPS is stacking-treated by the factory, which not only takes up land resources but also damages the environment and affects human health by dissolving the phosphorus and fluorine components in rainwater [5,6]. Therefore, it is essential and urgent to find ways to dispose of YPS.

Meanwhile, cement is one of the three basic materials in the construction industry and has high carbon emissions and energy consumption in its production [7]. In recent decades, CO_2_ emission reduction, environmental protection and energy conservation have been major concerns for the cement industry [8,9]. A large number of industrial solid wastes have been widely applied in cement and concrete as cementitious materials or mineral admixtures [10,11,12]. This not only reduces the cost of solid waste dam management and operation and cement production but also decreases the environmental pollution problems caused by solid waste storage [13].

The chemical composition of YPS varies depending on the raw materials and manufacturing process. Molten YPS is rapidly cooled by water quenching from high temperature to room temperature. CaO and SiO_2_ are the main components of YPS, and the glassy-phase content can reach 85–90% in YPS [14]. Therefore, YPS has potential pozzolanic activity and can be applied to the field of building materials such as cement, concrete, supplementary cementitious materials, road base materials and backfill materials [15,16]. A small amount of YPS replacing silicate cement in concrete can decrease the dosage of silicate cement. YPS reacts with calcium hydroxide in cement and produces C-S-H gel during cementitious reaction, which can refine the microstructure of concrete, reduce harmful pores and improve the mechanical properties of concrete. Wang et al. [17] investigated the impact of YPS on the durability and mechanical properties of concrete. The results indicated that the activity of YPS was greater than that of fly ash. The high-volume electric furnace nickel slag-YPS concrete showed similar or even better mechanical properties and chloride ion permeability than high-volume fly ash concrete at later stages. Yang et al. [18] found that adding YPS significantly improved the flowability and reduced the autogenous shrinkage in the early stage of ultrahigh performance concrete. Although it decreases the early compressive strength of ultrahigh performance concrete, it still promotes long-term strength development.

The strength of cement mortar at an early age decreases significantly with increasing YPS dosage, which restricts the utilization of YPS in cement and concrete [19]. The early activity of YPS is comparatively poor, and the fluorine and phosphorus in YPS will delay the hardening and setting of cement [20]. The phosphorus is uniformly distributed in YPS. The distribution pattern of P is very similar to that of Si. P is mainly present in the solid solution of 3CaO·P_2_O_5_ and 2CaO·SiO_2_ [21]. Therefore, it is vital to improve the pozzolanic activity of YPS and enhance the early strength of high-value YPS-based cementitious materials. Mechanical, chemical and thermal activation methods are applied to enhance the pozzolanic activity of YPS [22,23,24,25,26]. He et al. [27] noted that the slurry pH, activity index and uniformity of YPS particle sizes significantly increased with increasing YPS granularity, and the impact on the strength and setting time of cement paste was significantly reduced. Hu et al. [28] found that superfine YPS refined the microstructure of concrete, which helped to improve the resistance to chloride ion penetration and carbonation of concrete in the later stage. In addition, superfine YPS is helpful for the late development on the splitting tensile strength and compressive strength of concrete. Allahverdi et al. [29] reported that an improvement in the specific surface area of YPS with mechanical activation significantly reduced the amount of water absorption and the total open pore volume of mortars. The synergistic combination of mechanical activation and chemical activation can enhance the quality of high YPS cement. Additionally, chemically activated YPS-based composite cement has a superior resistance to sodium sulfate [30]. Zhang et al. [31] found that the application of YPS retarded the early hydration of cement, but increasing the curing temperature tended to decrease this retarding effect. Wang et al. [32] showed that adding YPS increased the late compressive strength of fly ash-based geopolymers. Increasing the curing temperature and NaOH concentration can improve the compressive strength of geopolymers. Heat treatment or high-temperature curing can increase strength development but may also reduce ultimate strength [33]. However, most of the scholars studied the effect of high-temperature curing on the properties of YPS-based cement. The effect of heat treatment on the pozzolanic activity and structural change in YPS has rarely been studied.

The aim of this study is to increase the pozzolanic activity of YPS by thermal activation. The strength method, alkali dissolution method and polymerization degree method were used to evaluate the effect of thermal activation at different temperatures on the pozzolanic activity of YPS. The Fourier-transform infrared spectrometry (FTIR) and X-ray diffraction (XRD) techniques were used to characterize the phases formed during the calcination process. This study explores the mechanism of YPS during calcination and lays the foundation for the large-scale utilization of YPS.

## 2. Materials and Methods

YPS was provided by Guizhou, China, and 42.5 grade cement was taken from the Hebei cement plant. The standard sand was obtained from the Xiamen sand plant. The NaOH (Aladdin) was an analytical grade chemical reagent. Table 1 shows the chemical compositions of the raw materials. The main components of both YPS and cement are CaO and SiO_2_, and less Al_2_O_3_.

**Figure 1:**
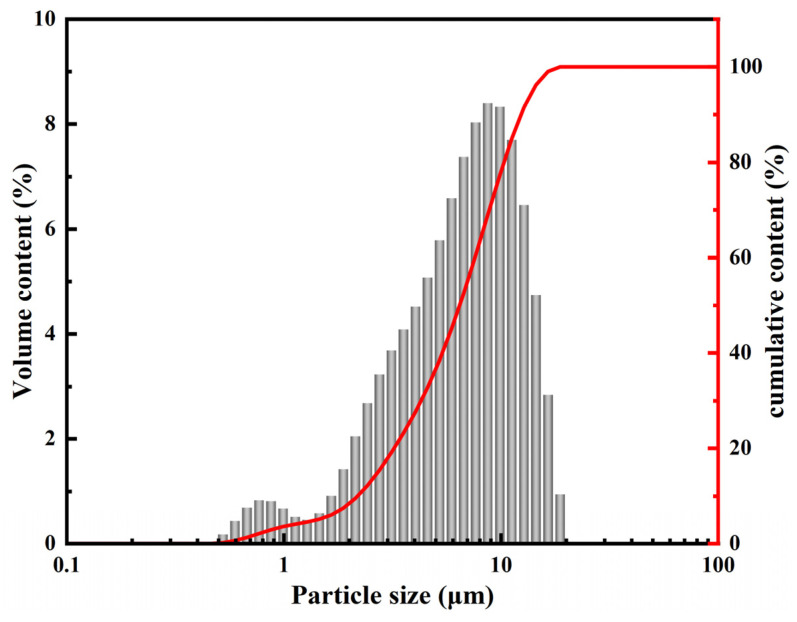
The particle size distribution of YPS.

First, YPS was dried in the oven and milled in a ball mill for 90 min. The specific surface area of the ground YPS measured by the FBT-9 full automation instrument testing specific surface was 421 m^2^/kg, and Figure 1 shows its particle size distribution tested by a laser particle size analyzer (Mastersizer 2000, Malvern Instruments Ltd., Malvern, UK). The ground YPS particles are within the range of 0.5–18.7 μm with a mean of 7.30 μm.

**Figure 2:**
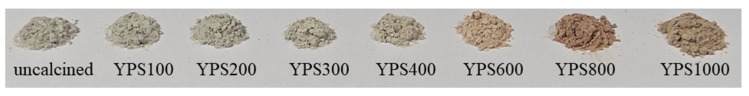
The visual attributes of YPS ash.

Second, 300 g of ground YPS was placed in a muffle furnace, calcined at 100–1000 °C for 3 h and then cooled to room temperature. The heating speed of the muffle furnace was 5 °C/min. The YPS ash calcined at 800 °C and 1000 °C required grinding in a small ball mill for 20 min. YPS (*n* = 100–1000) represents the YPS ash calcined at different temperatures. Figure 2 shows the visual attributes of YPS ash. The color of YPS ash gradually changed from gray-white to flesh pink with the increase in calcination temperature. Finally, the pozzolanic activity of YPS ash was tested by the strength method, alkali dissolution method and polymerization degree method.

The strength method was carried out according to the Chinese standard “ground granulated electric furnace phosphorus slag powder used for cement and concrete” (GB/T 26751-2022) [34]. The activity of YPS ash was evaluated by comparing the compressive strength of cement mortar with or without the addition of YPS ash. The specific steps were as follows: 225 g of water, 135 g of YPS ash, 315 g of cement and 1350 g of standard sand were mixed and stirred and then injected into a 40 × 40 × 160 cm mold with vibration; the mortar was put into a curing box with a temperature of 20 ± 1 °C and humidity of 95% for 3, 7 and 28 d. The proportion of the control group was 450 g of cement and 1350 g of standard sand. Table 2 shows the mix proportions of each sample. A press (HYE-300-10, Beijing Sanyulutong Instrument Co., Ltd., Beijing, China) was used to test the compressive strength of the mortar samples. The pozzolanic activity index (A) of YPS ash was evaluated according to Equation (1):

(1) $$A=\frac{R_{i}}{R_{0}}\times 100,$$

where *R_i_* (MPa) represents the compressive strength of the YPS sample and *R*_0_ (MPa) represents the compressive strength of the control sample.

The steps of the alkali dissolution method were as follows: 1 g of YPS ash was taken into 100 mL of 1 mol/L NaOH solution, sealed and put in a curing box at 20 ± 1 °C for 7 d; then, it was filtered to obtain the filtrate. An ICP-OES (Optima 7000DV, PerkinElmer Instrument Co., Ltd., Shanghai, China) was used to test the concentrations of Si and Al in the filtrate.

The polymerization degree method used Origin software to fit and calculate the peak area of Si(Al)Q^n^ at 800–1200 cm^−1^ of the FTIR spectra. Then, the polymerization degree of [SiO_4_] was evaluated using the concept of the relative bridging oxygen bond (RBO) according to Equation (2) [35]:

(2) $$\mathrm{RBO}=\frac{1}{4}\left(1\times \frac{Q^{1}}{\sum Q^{n}}+2\times \frac{Q^{2}}{\sum Q^{n}}+3\times \frac{Q^{3}}{\sum Q^{n}}+4\times \frac{Q^{4}}{\sum Q^{n}}\right)=\frac{1}{4}\times \frac{\sum n\times Q^{n}}{\sum Q^{n}}$$

The chemical compositions of the YPS ash and cement were tested by X-ray fluorescence spectrometry (Shimadzu XRF-1700 series, Shanghai, China). The phase composition of YPS ash at different calcination temperatures was analyzed by an X-ray diffractometer (D8 advance, Germany). The experimental conditions were Cu target, current 40 mA, voltage 40 kV, scanning speed of 10°/min and scanning range of 10–90°.

Infrared spectra of YPS ash calcined at different temperatures were analyzed by Fourier-transform infrared spectrometry (Nicolet IS10, Thermo Nicolet Corporation, Madison, GA, USA) using the KBr pellet technique. The specific steps were as follows: 200 mg of KBr and 2 mg of sample were mixed evenly, ground to less than 5 µm and made into transparent slices. The measured wavenumber range was 400–4000 cm^−1^.

## 3. Results

### 3.1. XRD Analysis

**Figure 3:**
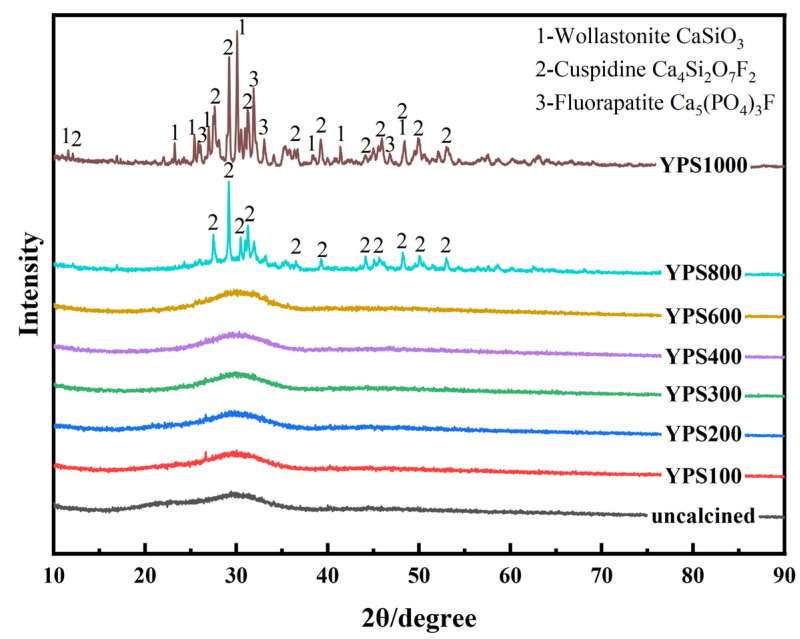
XRD patterns of YPS ash uncalcined and calcined at different temperatures.

YPS is a complex industrial waste whose chemical composition and mineral phase vary greatly depending on the process and the raw materials used in its production. The main chemical compositions of YPS used in this study are calcium, silicon and aluminum, as well as small amounts of phosphorus, fluorine, sulfur, magnesium, potassium and iron. The XRD spectra of YPS ash uncalcined and calcined at 100–1000 °C are shown in Figure 3. The XRD spectra of uncalcined YPS and YPS100-600 are similar. None of them have significant diffraction peaks, and there is a major broad peak at 20–40°. This indicates that the YPS samples under these conditions are low chemically stable glassy substances, which is favorable for the pozzolanic reactions.

The phase compositions of YPS ash change gradually with increasing temperature. In the XRD spectra of YPS800, the diffraction peaks of cuspidine (Ca_4_Si_2_O_7_F_2_) appeared with the presence of bun peaks, and the area of the bun peaks decreases. The XRD spectra of YPS1000 shows that the diffraction peaks of the different phases are sharp and clear, indicating that there is little amorphous content in YPS ash and that the crystallinity of the phases is high. As the calcination temperature is raised from 800 °C to 1000 °C, the wollastonite (CaSiO_3_) and fluorapatite (Ca_5_(PO_4_)_3_F) phase appears, and the diffraction peak of cuspidine in YPS ash is enhanced. The main phase compositions of YPS1000 are wollastonite, cuspidine and fluorapatite.

### 3.2. Pozzolanic Activity Evaluation by Compressive Strength

**Figure 4:**
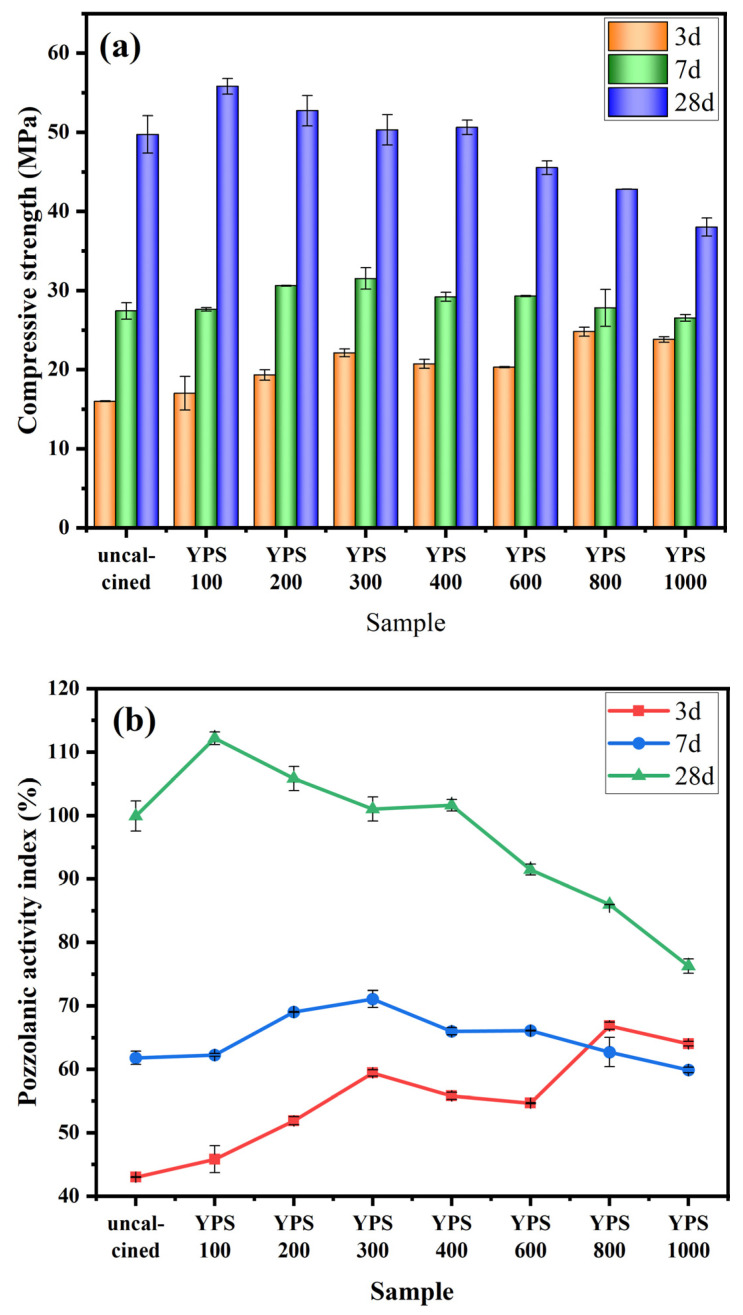
The (**a**) compressive strength of samples and (**b**) pozzolanic activity index of YPS ash uncalcined and calcined at different temperatures.

The pozzolanic activity of YPS ash can be calculated by the compressive strength of the YPS mortar. Figure 4 shows the compressive strength of YPS cement mortars and the pozzolanic activity index of YPS ash for 3, 7 and 28 d (the compressive strength of the control cement mortar is 37.1 MPa, 44.3 MPa and 49.8 MPa at 3, 7 and 28 d, respectively.). The compressive strength of the cement mortar varies with the calcination temperature, which proves that the calcination temperature has a significant effect on the cementitious properties of YPS ash. The compressive strength of YPS cement mortar probably increases and then reduces with the increasing calcination temperature. YPS800 has the highest 3 d compressive strength, with a value of 24.8 MPa, which is 55.0% higher than that of uncalcined YPS. This may be because the fluorine present in cuspidine is mainly insoluble and has little effect on the setting time of mortar [21]. However, the 28 d strength of YPS800 is relatively low, because the formation of crystalline phases makes the silica and aluminum in YPS800 difficult to dissolve at the later stage. This phenomenon illustrates that heat treatment can increase early strength but reduce ultimate strength. The 3 d compressive strength from YPS800 to YPS1000 decreases, which may be due to the generation of soluble Ca_5_(PO_4_)_3_F at 1000 °C, prolonging the setting time of the cement mortar and reducing its strength [21]. Compared to YPS uncalcined and YPS100-600, the 3 d compressive strength of YPS1000 is higher because a portion of fluorine in YPS1000 is present in the cuspidine, which reduces the retardation effect of fluorine on YPS1000 mortar. This also demonstrates that phosphorus and fluorine in the amorphous phase of YPS uncalcined and YPS100-600 are more easily soluble and affect the setting of YPS mortar. The 7 d compressive strength of the YPS300 mortar is the best, with a value of 31.5 MPa. The 28 d compressive strength of the YPS100 mortar is the highest, with a value of 55.8 MPa. This may be due to the gradual decrease in the amorphous substances in YPS with increasing calcination temperature.

As can be seen from Figure 4b, the change in the pozzolanic activity index of YPS uncalcined and calcined at different temperatures is the same as the change in its compressive strength. The calcination of YPS can improve the pozzolanic activity index at 3 d, but it will reduce the pozzolanic activity index at 28 d, and the enhancement of the pozzolanic activity index at 7 d is less obvious. The calcination of YPS at 800 °C is favorable to enhance the compressive strength of YPS cement mortar at an early age, while YPS calcined at 100 °C is conducive to increasing the ultimate strength of YPS cement mortar.

### 3.3. Pozzolanic Activity Evaluation by Dissolution Concentrations of Si and Al

Reactive Si and Al dissolved in alkaline environments can take part in the hydration reaction of cement. Therefore, the pozzolanic activity of YPS ash can be evaluated by the alkaline dissolution method. The dissolution concentrations of Si and Al in a 1 mol/L NaOH solution of YPS uncalcined and calcined at different temperatures are shown in Table 3. The dissolution concentrations of Si and Al first increase and then decrease with increasing temperature. The dissolution concentrations of Si and Al in YPS100 are 41.01 mg/L and 30.08 mg/L, respectively. Its dissolution concentration of (Si + Al) is the highest, which is consistent with the 28 d compressive strength of YPS cement mortars. The lowest dissolution concentrations of Si, Al and (Si + Al) are 7.197 mg/L, 10.08 mg/L and 17.28 mg/L in YPS1000, respectively. And its 28 d compressive strength and pozzolanic activity index at 28 d are also the lowest.

**Figure 5:**
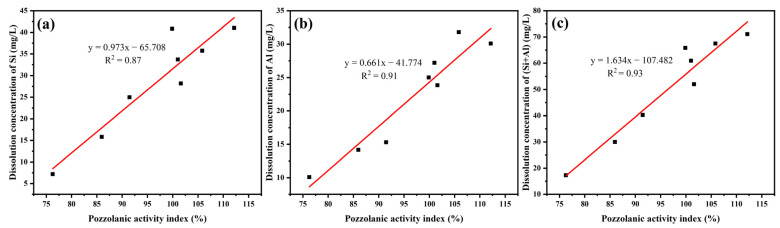
The relationship between the dissolution concentrations of (**a**) Si, (**b**) Al and (**c**) (Si + Al) and the pozzolanic activity index of YPS.

The trend of the pozzolanic activity index for 28 d with temperature is basically consistent with that of the dissolution concentrations of Si and Al. Therefore, the relationship between the pozzolanic activity index of YPS ash at 28 d and the dissolution concentrations of Si and Al has been studied, as shown in Figure 5. The dissolution concentrations of Si, Al and (Si + Al) in YPS ash are positively related to its pozzolanic activity index for 28 d. The pozzolanic activity index for 28 d increases with increasing dissolution concentrations of Si, Al and (Si + Al). The linear fit R^2^ about pozzolanic activity index and dissolution concentrations of Si, Al and (Si + Al) was progressively increasing with values of 0.87, 0.91 and 0.93, respectively. This shows that the dissolution concentrations of both Si and Al have an effect on the pozzolanic activity of YPS ash. The change rule of the pozzolanic activity index for 3 and 7 d and the dissolution concentration of Si and Al is a little different, which indicates that the early strength of YPS mortar is not only affected by the dissolution concentration of Si and Al but also by the role of soluble fluorine and phosphorus.

### 3.4. Pozzolanic Activity Evaluation by FTIR

**Figure 6:**
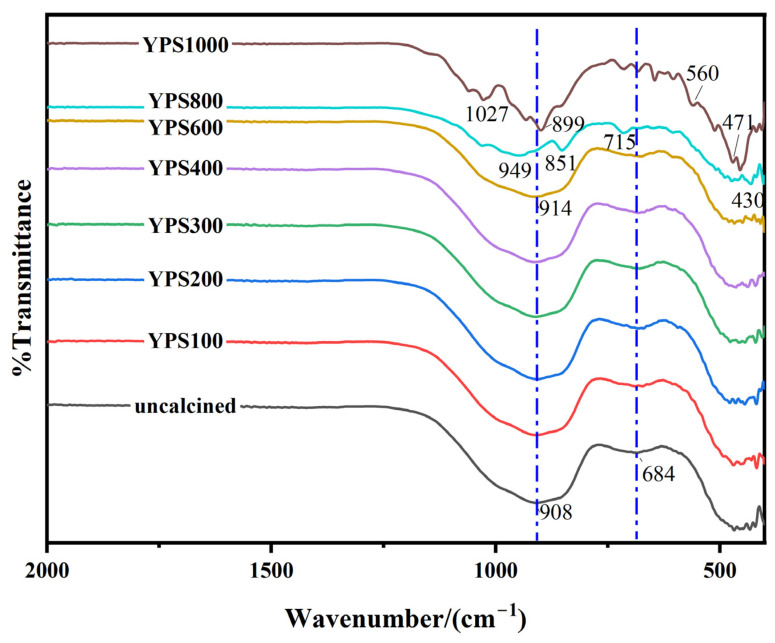
FTIR spectra of YPS ash uncalcined and calcined at different temperatures.

The FTIR spectra of YPS ash uncalcined and calcined at 100–1000 °C are shown in Figure 6. The FTIR spectra of YPS ash uncalcined and calcined in the range of 100–600 °C are very similar and show analogous absorption bands. The FTIR spectra can be divided into three regions of 800–1200 cm^−1^, 600–800 cm^−1^ and 400–600 cm^−1^. The peaks of 800–1200 cm^−1^ correspond to the asymmetric tensile vibrations of Si-O-(Si, Al) linked to the tetrahedral of [SiO_4_] or [AlO_4_]^−^ [32]. The peaks at 600–800 cm^−1^ are the symmetric stretching vibrations of Si-O-Al or Si-O-Si in the tetrahedra of [SiO_4_] or [AlO_4_]^−^ [35]. The peaks in the 400–600 cm^−1^ range are the bending vibrations of Si-O-Si(Al) [29,35]. The positions and areas of the peaks of 800–1200 cm^−1^ vary with temperature, indicating that the bonds between Si, Al and O in YPS ash are combined or broken. The peaks of the Si-O-Si/Al bonds move to higher wavenumbers (949 cm^−1^) with increasing temperature, indicating an improvement in the polymerization degree. The absorption peaks belong to PO_4_^3−^ bands at 471 cm^−1^, 560 cm^−1^ and 1027 cm^−1^, which proves the formation of fluorapatite at 1000 °C [36]. This is consistent with the results of the XRD analysis.

**Figure 7:**
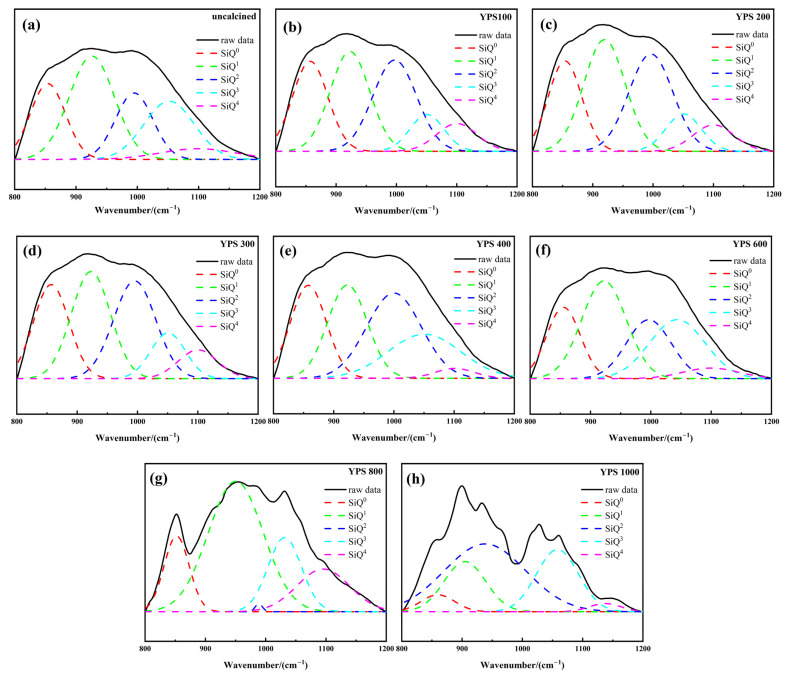
The fitted peaks in the range of 800–1200 cm^−1^ of YPS ash uncalcined and calcined at different temperatures: (**a**) uncalcined; (**b**) YPS100; (**c**) YPS200; (**d**) YPS300; (**e**) YPS400; (**f**) YPS600; (**g**) YPS800; (**h**) YPS1000.

The five bands at 1200 cm^−1^, 1050 cm^−1^, 1000 cm^−1^, 900 cm^−1^ and 850 cm^−1^ are assigned to Q^4^ Si, Q^3^ Si, Q^2^ Si, Q^1^ Si and Q^0^ Si, respectively. The superscript n is the number of bridge oxygens attached to each Si. The polymerization degree of the silicate structure is higher as n increases. The peaks between 800 and 1200 cm^−1^ were separated and fitted with Origin software to calculate the RBO. Figure 7 shows the fitted peaks between 800 and 1200 cm^−1^ of YPS ash uncalcined and calcined at different temperatures. The relevant parameters of the peaks are shown in Table 4. The polymerization degree of YPS ash first decreases and then increases with increasing temperature. The high degree of polymerization indicates that silicon and aluminum are relatively stable. The lower degree of polymerization demonstrates that the Si-O-Si(Al) in YPS ash is broken, and the active silicon and active aluminum in YPS ash are increased [35]. The RBO of the YPS100 is the smallest, with a value of 0.3757. The RBO of the YPS1000 is the biggest, with a value of 0.4982. This result is consistent with the 28 d compressive strength and dissolution concentrations of Si and Al. In addition, the R^2^ of the infrared fitting result decreases when the temperature is more than 800 °C. This is due to the appearance of a new crystalline phase, which can also be seen from the XRD results. Therefore, the RBO evaluation method is suitable for materials with no other influence in the range of 800–1200 cm^−1^.

**Figure 8:**
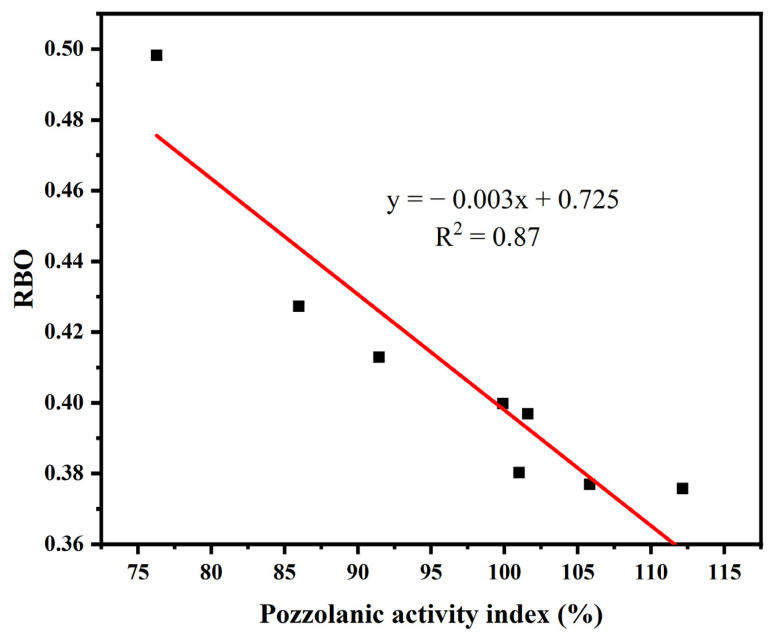
The relationship between RBO and the pozzolanic activity index of YPS ash.

**Figure 9:**
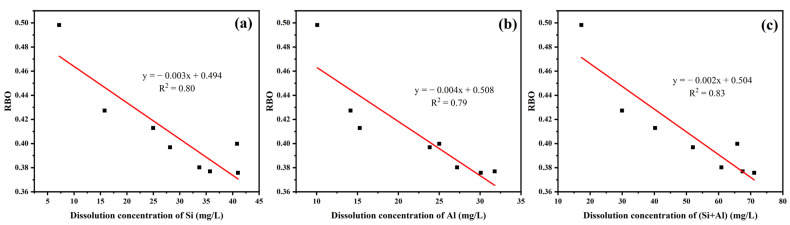
The relationship between RBO and the dissolution concentrations of (**a**) Si; (**b**) Al; and (**c**) (Si + Al).

Figure 8 shows the relationship between the pozzolanic activity and RBO of YPS ash uncalcined and calcined at different temperatures. The RBO of YPS ash tends to decrease with increasing pozzolanic activity. Figure 9 shows the relationship between the RBO and the dissolution concentrations of Si, Al and (Si + Al) of YPS ash uncalcined and calcined at different temperatures. RBO has a linear relationship with the dissolution concentrations of Si and Al. The decrease in RBO promotes an increase in the dissolution concentration of Si and Al in the YPS ash. The linear fit R^2^ about the RBO and pozzolanic activity is 0.87. The linear fits R^2^ about the RBO and dissolution concentrations of Si, Al and (Si + Al) are 0.80, 0.79 and 0.83, respectively. The linear fits R^2^ are poor compared with that of pozzolanic activity and dissolution concentrations of Si, Al and (Si + Al). This is mainly due to the fact that the infrared peaks in the range of 800–1200 cm^−1^ of YPS1000 are interfered with by other functional groups and the fitting effect of RBO is weakened. This shows that the dissolution concentrations of both Si and Al have an effect on the pozzolanic activity of YPS ash. The polymerization degree method for evaluating the pozzolanic activity of YPS ash takes less time and is more convenient compared to the strength and alkali dissolution methods, but it is susceptible to the influence of the physical phase.

## 4. Conclusions

In this paper, the phase and structural transformations of YPS ash during calcination at 100–1000 °C were investigated, and the changes in the pozzolanic activity of YPS ash were studied by the compressive strength method, alkaline dissolution method and polymerization degree method. This study provides insight into the gelling behavior of YPS ash.

The uncalcined YPS mainly contains amorphous aluminosilicates. As the calcination temperature increases to 800 °C, crystalline phases appear in the YPS ash. Cuspidine (Ca_4_Si_2_O_7_F_2_) is formed at 800 °C. The formation of fluorapatite (Ca_5_(PO_4_)_3_F) and wollastonite (CaSiO_3_) occurs at 1000 °C. The polymerization degree decreases and then increases with increasing calcination temperature. The polymerization degree is lowest at 100 °C. Due to the change in phase and polymerization degree, the calcination temperature has a great influence on the pozzolanic activity of YPS ash. It was found that YPS ash calcined at 800 °C favored the early strength of the sample, and YPS ash calcined at 100 °C had the highest 28 d compressive strength.

The relationships between the dissolution concentration of (Si + Al), compressive strength and polymerization degree and the pozzolanic activity of YPS ash were analyzed. The pozzolanic activity of YPS ash is positively correlated with the compressive strength and the dissolution concentration of (Si + Al) and negatively associated with the polymerization degree.

This study provides a possibility for the utilization of YPS in cement and concrete, which is conducive to the use of YPS in large dosages.

## Figures and Tables

**Table 1:** The chemical compositions of raw materials (wt.%).

Oxides	CaO	SiO_2_	Al_2_O_3_	Fe_2_O_3_	MgO	Na_2_O	K_2_O	SO_3_	P_2_O_5_	F
YPS	44.72	34.84	6.00	1.19	1.94	0.35	1.43	2.36	3.18	3.24
Cement	63.48	20.70	5.00	3.59	2.76	0.14	0.82	2.59	0.075	0.096

**Table 2:** The mix proportions of the sample.

Sample	YPS Ash/g	Cement/g	Standard Sand/g	Water/g	w/c Ratio
YPS sample	135	315	1350	225	0.5
Control sample	0	450	1350	225	0.5

**Table 3:** The dissolution concentrations of Si and Al in YPS ash uncalcined and calcined at different temperatures (mg/L).

Sample	Dissolution Concentration of Si	Dissolution Concentration of Al	Dissolution Concentration of (Si + Al)
uncalcined	40.83	25.01	65.84
YPS100	41.01	30.08	71.09
YPS200	35.71	31.79	67.50
YPS300	33.71	27.19	60.90
YPS400	28.17	23.84	52.01
YPS600	24.97	15.29	40.26
YPS800	15.79	14.16	29.95
YPS1000	7.197	10.08	17.28

**Table 4:** The relevant peaks parameters of YPS ash uncalcined and calcined at different temperatures.

Sample	Relative Content/%					RBO	R^2^
	SiQ^0^	SiQ^1^	SiQ^2^	SiQ^3^	SiQ^4^		
uncalcined	19.24	34.83	18.43	21.78	5.72	0.3998	0.996
YPS100	23.22	29.34	29.75	9.33	8.36	0.3757	0.996
YPS200	20.63	32.40	30.14	9.24	7.59	0.3769	0.997
YPS300	22.75	29.38	28.86	11.07	7.94	0.3802	0.997
YPS400	21.48	24.35	30.66	20.95	2.56	0.3969	0.996
YPS600	17.78	33.02	19.95	24.81	4.44	0.4128	0.997
YPS800	13.68	51.86	0.42	17.95	16.09	0.4273	0.983
YPS1000	5.14	19.10	49.46	23.94	2.36	0.4982	0.963

## Data Availability

Data sharing is not applicable to this article.
